# Success in publication by graduate students in psychiatry in Brazil: an empirical evaluation of the relative influence of English proficiency and advisor expertise

**DOI:** 10.1186/1472-6920-14-238

**Published:** 2014-11-06

**Authors:** Alexandre Cunha, Bernardo dos Santos, Álvaro Machado Dias, Anna Maria Carmagnani, Beny Lafer, Geraldo F Busatto

**Affiliations:** Department of Psychiatry, University of São Paulo School of Medicine, São Paulo, Brazil; Núcleo de Apoio à Pesquisa em Neurociência Aplicada (NAPNA, Center for the Support of Research in Neuroscience), University of São Paulo, São Paulo, Brazil; Institute of Mathematics and Statistics, University of São Paulo, São Paulo, Brazil; Laboratory of Clinical Neuroscience, Universidade Federal de São Paulo (UNIFESP, Federal University of São Paulo), São Paulo, Brazil; English Department, School of Philosophy, Literature, and the Human Sciences, University of São Paulo, São Paulo, Brazil

**Keywords:** Knowledge of English, Publication in English, Language, Translation, Publishing, Journal impact factor, Psychiatry

## Abstract

**Background:**

This study evaluates the success of graduate students in psychiatry in an emerging country, in terms of the quantity and quality of their publication productivity (given by the number of papers and impact factors of the journals in which they publish). We investigated to what extent student proficiency in English and the scientific capabilities of academic advisors predict that success.

**Methods:**

Our sample comprised 43 master’s and doctoral students in psychiatry (n = 28 and n = 15, respectively) at the University of São Paulo School of Medicine, in São Paulo, Brazil. We collected information about their knowledge of English and the ways in which they wrote their articles to be submitted to periodicals published in English. Multiple regression analyses were carried out in order to investigate the influence English proficiency, h-index of supervisors and use of language editing assistance had on the number and impact of student publications.

**Results:**

Although 60% of students scored ≥80 (out of 100) on English tests given at admission to the graduate program, 93.09% of the sample used some form of external editing assistance to produce their papers in English. The variables “number of publications” and “impact factor of journals” were significantly related to each other (r = 0.550, p < 0.001). Multiple regression analysis revealed that the impact factor of periodicals where students published their articles as first authors correlated significantly not only with student proficiency in English at admission (p = 0.035), but also with the degree of language editing assistance (p = 0.050) and the h-index of the academic advisor (p = 0.050).

**Conclusions:**

Albeit relevant, knowledge of English was not the key factor for the publication success of the graduate students evaluated. Other variables (h-index of the advisor and third-party language editing assistance) appear to be also important predictors of success in publication.

**Electronic supplementary material:**

The online version of this article (doi:10.1186/1472-6920-14-238) contains supplementary material, which is available to authorized users.

## Background

English is undisputedly the leading international language for academic communication and other professional purposes [1].

The issues involved in the publication of research articles in English as a second language have been extensively investigated for more than two decades [2]. Qualitative studies evaluating individuals who use English as a second language have suggested the existence of a direct link between success in publication and level of proficiency in English [3–5]. In addition, qualitative investigations in the fields of applied linguistics and literacy education have highlighted the influence of other variables in the process of successfully publishing in English as a second language, including educational and cultural backgrounds, types of academic and social networks, persistence and other psychological processes, scientific training, editorial bias, global developments in the usage of English, geographical location and socio-economic resources [6–9].

Conversely, quantitative research investigations in this field have been scarce, and only a few studies have evaluated statistically the relationship between the above variables and the degree of academic success [10, 11].

The impact of a scientific journal, as measured by indices such as the average number of citations given to recently published articles, is frequently used to indicate the influence that a given periodical exerts in its field [12]. Publication of research results in scientific journals of high impact is often taken to measure the degree of academic success in Western academic institutions, and the overwhelming majority of high-impact scientific journals are published in English. In recent years, the aim of publishing research articles in periodicals of higher impact has become increasingly relevant also in mid-income countries outside North America and Europe, including Brazil, China and India [13]. Academics who cannot master their writing expression in English put their careers as well as the science they produce at stake.

One notable area of biomedical knowledge is that of neuroscience and psychiatry. In countries like Brazil, while the annual number of publications by authors working in the area of psychiatry has remained stable, these articles have appeared more often in journals of higher impact [14]. Graduate programs in Brazil are nowadays classified and funded by the federal government based on a ranking that gives the greatest weight to the number of publications by students and their supervisors in peer-reviewed periodicals, as well as the impact factor of such periodicals as measured by the Institute for Scientific Information (ISI) [15].

To our knowledge, there have been no quantitative research studies investigating the relationship between proficiency in English and scientific productivity specifically in the field of psychiatry in non-English speaking countries. Graduate programs are interesting settings for this type of investigation, because graduate students are less experienced compared to established researchers, and baseline proficiency in English can therefore be presumed to have a more decisive influence on the acceptance of their manuscripts for publication in journals of higher impact. In addition, quantitative studies of publication in English allow the statistical analysis not only of the objective measurement of student proficiency in English but also of other factors, such as the ability of the academic advisors themselves to publish in such journals and student access to translating and language editing assistance. The number and impact of the articles published by a researcher are of ever-increasing value for evaluating and rating graduate programs in emerging countries. Therefore, studies of this kind can help to inform decisions regarding the management of graduate programs in countries such as Brazil.

The present study aimed to evaluate the success of graduate students in psychiatry in Brazil, in terms of the quantity and quality of their publication productivity, investigating the influence that student proficiency in English and the scientific capabilities of academic advisors (in terms of the quantity and impact of their papers published in indexed, peer-reviewed periodicals) have on student ability to publish. We hypothesized that both student proficiency in English and the scientific competence of academic advisors would be predictive of the ability of students to publish.

## Methods

### Participants and procedures

All (n = 46) graduate students (master’s and doctoral students) who obtained their degrees from the Department of Psychiatry of the *Faculdade de Medicina da Universidade de São Paulo* (FMUSP, University of São Paulo Medical School) between July 2009 and May 2011 were invited to take part in the study, and 43 of them agreed to participate. In the program for Masters and PhD degree courses at this department, the criterion for eligibility includes a degree in Medicine, Psychology or other biomedical specialties. A significant proportion of the physicians who enter this program have previously undergone medical residency in Psychiatry, but this is not a prerequisite for entry. It is worth noting that the obtainment of a Masters or PhD degree is not a requirement for physicians or psychologists to practice in the Brazilian system. The FMUSP Graduate Program in Psychiatry has objective rules regarding the publication of research results: students enrolled in the program can defend their theses only after they have submitted their results for publication in a journal indexed by the ISI; and students cannot be awarded a doctorate if their results have not been accepted for publication in an ISI-indexed journal. Periodically (every three years), academic advisors are also evaluated in terms of the quantity and quality of their publication productivity, as given by number of published articles in ISI-indexed periodicals and the impact factor of these journals. The program offers professional translation services, at no charge, to every student enrolled in the program if requested.

The subjects were invited to complete an online questionnaire, comprising 32 questions, in three sections (see Additional file 1): Part A (consisting of 9 multiple-choice questions) explores previous exposure to the English language; Part B (consisting of 7 questions) solicits a self-evaluation of the student’s knowledge of English; and Part C (consisting of 16 questions) collects information regarding the number of publications, the degree of difficulty that students have in preparing, submitting, and revising the first article(s) gleaned from their data, and the measures taken to overcome the obstacles encountered during the process (such as obtaining assistance to translate and/or edit their articles into proper English). The questions in Parts A and B were adapted from Vasconcelos et al. [10], whereas Part C was prepared specifically for use in the present study.

We obtained, from the admissions office of the FMUSP Graduate Program in Psychiatry, the scores on the English proficiency tests presented by students for enrollment in the program. A total of 41 subjects had undergone a test prepared specifically for candidates to the FMUSP graduate programs by a traditional English-language school from the state of São Paulo, referred to as “*Cultura Inglesa*” (maximum score being 100). The two remaining individuals had performed the Test of English as a Foreign Language (TOEFL), and their scores on that test had been used to warrant their admission at the FMUSP Graduate Program in Psychiatry. In order to make the ratings for the two types of English knowledge tests comparable, the TOEFL scores were converted to a 1-100 range by cross-multiplication. Each subject was assigned a number, and this number was used as a variable in both the spreadsheet for the questionnaire answers and the spreadsheet for English test scores, filled by the admissions office of the FMUSP Graduate Program in Psychiatry. The data file merging those two spreadsheets omitted subject’s names, in order to preserve their anonymity.

From the responses given on Part C of the questionnaire, we obtained the total number of articles related to the theses/dissertations accepted for publication in ISI-indexed journals as of May of 2012. The information was confirmed and augmented as necessary on the basis of data available on the websites of the ISI and PubMed. The impact factors of those journals were also obtained from the ISI website. As an objective measure of the scientific competence of the advisors, we obtained their h-index, defined as the number of papers authored by a researcher that have received a total number of citations higher or equal to h [16]. This index was obtained as of May of 2012, again via the ISI website.

This study was approved by the official ethics committee of FMUSP (‘Comissão de Ética para Análise de Projetos de Pesquisa – HCFMUSP’, protocol number 0681/10). All participants provided written consent after being informed of the details of the study.

### Statistical analysis

We performed the statistical analysis of the data in two stages. The first stage, descriptive analysis, consisted of tabulation of the self-reported level of English proficiency, the average English test score at enrollment in the graduate program, the rates of partial or total third-party translation of articles, the total numbers of articles accepted for publication in ISI-indexed journals, the impact factors of those journals, and the h-indices of the advisors. In the second stage, two multiple regression analyses were carried out in order to investigate the influence that English proficiency and other factors have on the number and impact of student publications, respectively using as dependent variables: the total number of articles published by students as first authors, and the ISI-based impact factors of the journals in which these articles were published. We decided to specify two multiple regression analyses at the outset of the investigation, since those two outcome measures of publication evaluate different aspects, respectively: quantity of articles and quality of periodicals (and, therefore, expected visibility of articles). For the multiple regression analysis using the total number of published articles as dependent variable, we used a model of Poisson regression for count data and a linear regression model for the analyses that evaluated the journal impact factors as dependent variable. In both cases, the independent variables were the average English test score at enrollment, the self-reported level of overall English proficiency (from 1 = poor to 5 = excellent), the degree of third-party assistance in editing/translating articles, and the h-index of the advisor. The variable ‘degree of third-party assistance in editing/translating articles’ had the following levels: 0 = no assistance required; 1 = revision of a final version written by the student in English; 2 = revision of a preliminary version written by student in English; 3 = full translation of a final version written in Portuguese by the student. We estimated the degree of correlation between English proficiency at enrollment and the self-reported level of English proficiency with the Pearson correlation coefficient. To determine whether third-party edition/translation of articles was associated with the average English test score at enrollment or with the self-reported level of English proficiency, we used biserial correlation. We set the type I error rate at 5%, and all tests were two-tailed. For the statistical analyses, we used Statistical Package for the Social Sciences, version 14.0 (SPSS Inc., Chicago, IL, USA) [17].

## Results

Descriptive statistics for the total sample evaluated (n = 43) are provided in Table 1, including 28 master’s students and 15 doctoral students. The overall level of previous knowledge of English was relatively high, not only in terms of the self-reported level of English proficiency but also in terms of the mean English test score at enrollment (79.12, standard deviation [SD]: 11.1), 60% of the subjects having scored ≥80 (out of 100). There were modest to moderate significant linear correlations between the average English test score at enrollment and the self-reported level of English proficiency, both in reading (*r* = 0.548; p < 0.001), writing (*r* = 0.392; p = 0.009) and total self-ratings (r = 0.351, p =0.019). The variables number of publications and impact factor of journals were significantly related to each other (r = 0.550, p < 0.001).

**Table 1:** **Descriptive statistics regarding English proficiency**, **scientific productivity of students and capability of supervisors**

	Minimum	Maximum	Mean	SD
Number of articles published as first author	0	2	0.88	0.68
Mean impact factor articles published as first author	0	12.7	1.79	2.44
Self-reported overall level of English	2.13	4.25	2.84	0.57
Self-reported writing level of English	1	4	3.25	0.72
Self-reported reading level of English	2	5	2.09	0.83
English test score at enrollment	50	96	79.12	11.10
H-index of supervisors	3	33	13.09	7.04

The average h-index of supervisors is also provided in Table 1. There was no significant correlation between the h-index of supervisors and English test scores of students (r = -0.013, p = 0.53).

Descriptive statistics regarding to the degree of provision of language editing assistance to students is shown in Table 2. Of the 43 subjects evaluated, 40 (93.09%) requested at least some degree of assistance in finalizing their articles in English, despite their familiarity with the language. However, only 5 students (11.62%) availed themselves of translation assistance for the entire article previously written fully in Portuguese. Across the entire sample, the assistance to students in editing/translating articles was provided by different sources, including: professional paid translators (n = 9); the official supervisor (n = 12); other Brazilian co-authors of the article (n = 7); international scientific collaborators (n = 3); the Brazilian supervisor followed by a professional translator (n = 3); the supervisor plus other Brazilian co-author followed by a professional translator (n = 4) and a Brazilian proof reader proficient in English (n = 2). Thus the use of professional translators was requested by a minority of subjects (n = 16).

**Table 2:** **Language editing assistance provided to students**

Third-party language editing assistance	Frequency	Percent	Valid percent
No translation	4	9.30	9.52
Final version revision	23	53.48	54.76
Preliminary version revision	10	23.25	23.80
Full translation	5	11.62	11.90
Valid total	42	97.67	100.0
No provided information	1	2.32	
Total	43	100.0	

We found that the variable “degree of third-party assistance in editing/translating articles” did not correlate with the average English test score at enrollment (F =0.289, p =0.833), nor with the self-reported level of English proficiency, either in reading (F =1.081, p =0.369) or writing (F =0.297, p =0.827).

During the study period, the subjects evaluated were listed as authors on an average of 1.93 articles (SD: 1), being the first authors on an average of 0.88 articles (SD: 0.68) (Table 1). Articles reporting original data accounted for an average of 1.72 articles (SD: 0.7) having the students listed as authors. Additional file 2 provides the full list of ISI-indexed journals where articles were published, the number of authors in each article, and the language used by these journals. The variables referring to the number of articles published by each student and the mean impact factor of the journals where their papers were accepted were not correlated to each other (r = -0.027, p = 0.865).

Table 3 provides the outcomes of the multiple regression analysis that had, as dependent variable, the total number of articles published by subjects as first authors. This analysis did not show any significant results (likelihood ratio = 7.395; p = 0.495), indicating that neither English proficiency nor the scientific abilities of the advisor were determinant to the amount of papers produced by subjects from their graduate work.

**Table 3:** **Descriptive statistics regarding language editing assistance provided to students**

	B	SE	z	p	RR
Intercept	-0.787	1.577	-0.499	0.618	0.455
Self-reported proficiency					
Overall	-0.103	0.584	-0.176	0.860	0.902
Writing	-0.585	0.428	-1.369	0.171	0.557
Reading	0.288	0.357	0.807	0.420	1.334
English test score at enrollment	0.013	0.020	0.666	0.506	1.013
H-index	0.022	0.027	0.807	0.419	1.022
Third party translation				0.321	
No translation	0.000				
Final version revision	-0.153	0.669	-0.229	0.819	0.858
Preliminary version revision	0.515	0.674	0.764	0.445	1.673
Full translation	-0.262	0.882	-0.297	0.766	0.769
McFadden pseudo-R^2^ =0.079					

Dependent: number of articles published as first author.

Table 4 provides the outcomes of the multiple regression analysis that had, as dependent variable, the mean impact of the journals in which these articles were published. This analysis showed that the impact of the journals where students published their articles was predicted by the average English test score at enrollment (p = 0.035), as well as the h-index of the advisor (p = 0.050), and the degree to which third-party translation assistance was requested (p = 0.050).

**Table 4:** **Multiple regression analysis with the mean impact factor of the journals where subjects published their papers as dependent variable**

Mean impact factor	B	SE	t	p	η ^2^parcial
Intercept	-3.871	3.089	-1.253	0.219	0.045
Self-reported proficiency					
Overall	1.049	1.171	0.895	0.377	0.024
Writing	-0.869	0.774	-1.123	0.269	0.037
Reading	-1.118	0.773	-1.445	0.158	0.060
English test score at enrollment	0.079	0.036	2.195	0.035	0.127
H-Index	0.121	0.053	2.269	0.030	0.135
Third party translation				0.050	0.208
No translation	0.000				
Final version revision	0.452	1.222	0.370	0.714	0.004
Preliminary version revision	2.715	1.332	2.039	0.050	0.112
Full translation	2.400	1.669	1.438	0.160	0.059
R^2^ =0.402					

## Discussion

By evaluating a group of students from a psychiatry graduate program in a non-English speaking country, this study aimed to investigate the inter-relationship between student proficiency in English, scientific capability of academic advisors, and quality and quantity of research articles produced by students under their advisors’ supervision.

The students evaluated showed a relatively high level of proficiency in English (60% of the students scoring ≥80), and their English test scores at enrollment were comparable to the self-reported levels of English proficiency. The good knowledge of English in our sample is probably attributable to the fact that students in large Brazilian cities such as São Paulo often seek specialized language schools to improve their English skills during high-school or undergraduate university courses, as well as to the fact that USP is the highest-ranked university in Brazil and one of the highest-ranked universities from emerging countries in the world [18]. The modest to moderate significant linear correlations between the two types of proficiency measures in our study suggests that our subjects had a reasonable self-perception of their ability to use the English language.

Despite the fact that the subjects in our sample had considerable previous knowledge of English, most of them sought at least some degree of English editing assistance. Such finding may be due to the fact that successfully publishing in journals of greater impact requires knowledge of scientific writing in English that was not covered in the English courses the students had taken previously. However, other factors may also be involved: some authors with excellent English skills may still seek editing on every of their documents, while others with poor English may resist looking for such assistance for reasons such as the payment that may be needed for such task (although our subjects were offered professional translation services at no charge by the Graduate program). Regardless of the variety of potential explanations for the high frequency of English editing assistance in the present study, one should bear in mind that these considerations may be valid for universities with the characteristics seen at USP in Brazil, and may not be generalizable to explain the behavior of students and researchers of institutions, nationalities and disciplines distinct from those evaluated herein.

Our multiple regression analyses did show that the English test score at enrollment had a significant influence on student success in publishing, as we hypothesized. It should be noted that such influence was related only to the impact factors of the journals in which the articles were published (and not to the total number of articles). This pattern of results suggests that English proficiency has a greater influence on the quality than on the quantity of papers published by students [19–21]. However, such claim has to be made with caution, since only one student managed to publish findings in a top ranked periodical, with an impact factor above ten (see Additional file 2); moreover, the mean impact factor of journals where the sample achieved publication was not high (less than two). This highlights the challenges of scientists in emerging countries to get their findings published in high-impact periodicals [13], and underscores the fact that several issues other than English proficiency influence on such kind of achievement.

We identified a direct relationship between the impact factors of the journals in which subjects published their articles and the scientific ability of their advisors, as measured by their h-indices. To our knowledge, this is the first study to demonstrate such an association in the area of psychiatry in an emerging country. Thus the addition of student proficiency in English and ability of advisors seems predictive of greater success in publication in our sample. One possible explanation for such combined pattern could be that students who are more proficient in English and more motivated to pursue a scientific career seek advisors that are more familiar with the processes involved in publishing articles in international journals. This possibility, however, is not directly supported by our data, since we found no statistically significant correlation between the h-index of supervisors and English test scores of students. One alternative interpretation is that advisors who have a higher h-index (and are arguably more familiar with the process of successfully publishing in international journals) seek students who they perceive as more likely to publish successfully (because of their English proficiency and other characteristics – the latter ones yet to be identified and measured).

It is also relevant to note that the assistance of professional translators was used by a minority of subjects (n = 16), while the additional sources of assistance for students to improve the English quality of their papers were quite varied (including the official supervisor, co-authors and international collaborators of the group). Consistent with that, most of the research papers evaluated herein were authored by more researchers than the students and their senior advisors (see Additional file 2). This suggests that the network of people available to interact with students when they arrive at knowledgeable research groups (including both local and international collaborators), may help to propel such young researchers to achieve success in their publications. Therefore, publication success or failure may be the consequence of the skills of more scientists than the student and their official supervisor.

As previously mentioned, the setting in which this study was carried out presented characteristics favorable to the investigation of success in publishing by graduate students in psychiatry: the students enrolled in the FMUSP Graduate Program in Psychiatry are highly motivated to publish their results in higher-impact journals; the program has objective rules for the publication of results by graduate students; and financial support is provided for translation of articles into English. However, those characteristics might limit the generalizability of our results. Therefore, our results await replication using student samples from other graduate programs at different universities.

Despite the novelty of the findings presented herein, their interpretation should be made with caution due to a number of other issues. First, our study design did not allow the distinction between language proficiency and other intellectual and psychological skills of the scientists; it is conceivable that young researchers who are more proficient than their peers in English have also a better previous scientific training or are also better in other intellectual domains that are instrumental to becoming a successful researcher, such as creativity or logical thinking [2]. Second, the way in which our data was collected does not allow us to determine whether the advantage gained by the students who were more proficient in English is related to their specific knowledge of the foreign language or to a broader linguistic issue (for instance, better comprehension and expression of verbal material in any language, which would certainly increase the chances of publishing a scientific manuscript) [6]. Third, according to multi-dimensional models of acculturation, language proficiency is seen as only one of the several variables involved, with the outcome of the acculturation process (e.g., full biculturalism) varying depending also on internalization of cognitive structures and perceptual processing [22]. Therefore, future studies might add measures of familiarity and identification with American and/or British cultures with the aim of increasing the predictive power of the quantity and quality of scientific publications of the individuals undergoing Masters and PhD degree courses in emerging countries [23]. Fourth, we did not evaluate in a quantitative fashion the several other variables that are also thought to influence the process of successfully publishing in English as a second language, including social networks, emotional issues and overall economic resources [1, 2, 8]. Further quantitative studies in settings as ours should attempt to control for the influence of those additional variables. Moreover, replication of the present findings is warranted in other emerging countries using samples of graduate students from diverse cultural, social and economic environments.

Finally, it is relevant to highlight that we presented herein data from 43 out of the 46 graduate students who defended their theses and dissertations over the period covered by our investigation. Therefore, we were able to reach almost the totality of students within the FMUSP Graduate Program in Psychiatry for that period. However, although the results may be taken as representative of the target population, it is important to acknowledge that the sample size was relatively small. One other potential limitation of our study is the fact that we used the impact factor of scientific journals as outcome measure, since this index is essentially aimed to provide a measure of quality of journals rather than to evaluate the productivity of individual scientists [19].

## Conclusions

The present study demonstrated that knowledge of English is not the key factor for the publication success of graduate students. Other variables (h-index of the advisor and third-party language editing assistance) appear to be also important predictors of success in publication. These results raise important policy issues for graduate programs in psychiatry, and possibly for those in neurosciences in general, in emerging countries. There have been suggestions that public universities in emerging countries should provide translation services to facilitate global dissemination of local research findings [13]. In the present study, the vast majority of the students who were found to be proficient in English indeed required some degree of language editing services. This finding might suggest that, in order to be competitive in terms of scientific production, graduate programs in emerging countries should invest in providing financial support for the translation of articles authored by its students, even if those students are known to be relatively proficient in English. One could argue that, in terms of short-term cost-effectiveness, such programs would be better served by facilitating student access to translation services than by investing in increasing student proficiency in English. However, recent studies suggest that other alternatives may be effective to improve scientists’ skills for publishing in English as a second language, including: short-term specialized courses on scientific English provided by native English speakers [8, 9]; collaborating-colleague workshops [7]; and the tutored use of copying as a legitimate form of learning how to write scientifically [24]. Future studies should address the comparative short-term and medium-term efficacy of such varied strategies in graduate services in emerging countries.

## Electronic supplementary material

Additional file 1: **Questionnaire.** (PDF 357 KB)

Additional file 2: **List of ISI-indexed journals where students’ articles were published.** (PDF 100 KB)
