# Supplementary material for: Success in publication by graduate students in psychiatry in Brazil: an empirical evaluation of the relative influence of English proficiency and advisor expertise
Source: BMC Med Educ. 2014 Nov 6;14:238. doi: 10.1186/1472-6920-14-238 (PMC4289391; doi:10.1186/1472-6920-14-238)
Supplement: Supplementary file 1 — Additional file 1: Questionnaire. (PDF 357 KB) [file 12909_2013_1058_MOESM1_ESM.pdf]

- APPENDIX 1 -

Questionnaire

- Part A –

1. **You became aware of the English language existence:**

As a child	
As a teenager	
As an adult	

2. **You started learning English:**

Before the age of 10	
Before the age of 20	
Before the age of 30	
After your 30's	

3. **As for your parents:**

They were both fluent in the English Language.	
Neither one of them had full knowledge of the English Language.	
Only your mother was fluent in the English language.	
Only your father was fluent in the English language.	

4. **Regarding your parents' education:**

Both had a bachelor's degree or higher level of education.	
Neither one had a bachelor's degree or higher level of education.	
Only your mother had a bachelor's degree or higher level of education.	
Only your father had a bachelor's degree or higher level of education.	

5. **Your English language level of education comes from:**

Only a mandatory subject taught at your official schools.	
Subjects taught at your official schools, as well as English language schools.	
Only English language schools.	
Neither from subjects taught at your official schools, nor from English language schools.	

6. **In your household:**

Both your parents considered it was important to learn the English language.	
Only your mother considered it was important to learn the English language.	
Only your father considered it was important to learn the English language.	
Neither one of your parents considered it was important to learn the English language.	

7. **The reason you studied English was:**

To take English tests at your official school.	
To take English tests at your official school, as well as to take tests at the English Language school.	
To take tests at the English language school only.	
You never did study English.	

8. **Regarding the English language:**

You wished to understand the lyrics in the English songs.	
You wished to speak the English language.	
You wished to read English texts.	
You never had the desire, or the curiosity to learn the English language.	

9. Have you ever attended a more specific English course (i.e. focused in scientific writing, basic conversation, etc.)?

No, I have never attended any specific English courses.	
Yes, I have attended a full, live in class specific course.	
Yes, I have attended a full online specific course.	
Yes, I have attended up to the basic level of a specific, live in class course.	
Yes, I have attended up to the basic level of a specific online course.	
Yes, I have attended up to the intermediate level of a specific, live in class course.	
Yes, I have attended up to the intermediate level of a specific online course.	
Yes, I have attended a specific, live in class course; however I stopped at the advanced level.	
Yes, I have attended a specific online course; however I stopped at the advanced level.	

- Part B -

1. Which of the following areas, would you currently consider most difficult?

Reading	
Writing	
Verbal Communication	
Oral Comprehension	

2. Which of the following areas, would you currently feel most comfortable with?

Reading	
Writing	
Verbal Communication	
Oral Comprehension	

3. How would you rate your current English reading ability

Excellent	
Very good	
Good	
Fair	
Poor	
Can not evaluate	

4. How would you rate your current English writing ability?

Excellent	
Very good	

Good	
Fair	
Poor	
Can not evaluate	

5. How would you rate your current English verbal communication ability?

Excellent	
Very good	
Good	
Fair	
Poor	
Can not evaluate	

6. How would you rate your current English oral comprehension ability?

Excellent	
Very good	
Good	
Fair	
Poor	
Can not evaluate	

7. Overall and regardless of the language (English or Portuguese), would you consider yourself a good writer? Please, specify.

- Part C -

1. **How many and which, scientific articles, in English, related to your thesis (dissertation) have been submitted, so far for publication in international journals? Please mention all the articles, regardless if they have or have not yet, been accepted for publication.**

Answers: _______________________________________

2. What was your English language, level of knowledge, at the time that you wrote your first scientific article for your thesis?

Excellent	
Very good	
Good	
Fair	
Poor	
Can not evaluate	

3**. What level of difficulty did you face, while writing the first scientific article in English related to your thesis (dissertation)?**

Answers: ____

4. What kind of assistance did you have, while translating your first scientific article related to your thesis (dissertation), into the English language?

No assistance	
Translator, proficient in English	
Coauthor, proficient in English	
International collaborator	

Thesis (dissertation) advisor	
Other:____________________	

5. If there was assistance in the English translation, of the first scientific article related to your thesis (dissertation), in which stage did it occur?

Correction of the final version issued in English by the researcher himself.	
Correction of the initial version issued in English by the researcher himself.	
Full translation process into the English language	
Other: _______________________________________	

6. If there was a revision made to the first scientific article in English, related to your thesis (dissertation), and not performed by you, this revision was handled by:

A language editing service company	
A professional editor / non – English native proofreader	
A professional editor / English native proofreader	
A general English professor	
An academics' colleague whose first or second language is English	

7 . After being submitted for publication, specify the history behind your first scientific article, in English, related to your thesis/ dissertation (accepted or not when first submitted, re-submitted or not into a different journal).

Answers: ___________________________________

8. Were there any language issues or discrepancies, ever mentioned by the unknown proofreaders or editor of the journal, regarding your first scientific

article in English, related to your thesis (dissertation)? If so, in your opinion, why were this language issues brought up?

Answers: ________________________________

9. In case there were difficulties in the acceptance of your first scientific article in English for international publication, do you believe there could have been other factors that influenced in this decision, none related to the language or the article's scientific content?

Answers:

10. After the experience of your first scientific article in English, related to your thesis (dissertation), would you consider that your level of English knowledge:

Remained the same	
Improved	
Worsened	

11. If you consider that your level of English knowledge has improved, what would you attribute this improvement to?

Answers:

12. After your first scientific article for the thesis (dissertation), would you consider, that there was better acceptance from the mediators, in regards to the written language? Describe:

13. How would you assess your progress, in the following articles to the first article for the thesis (dissertation)? Was the process more elaborated, or were there more support resources used? Specify:

14. **If you also publish in Portuguese (if not, move onto the next question), how many and which, scientific articles, related to your thesis (dissertation) have been submitted, so far for publication in national journals? Please, mention all the articles, regardless if they have or have not yet, been accepted for publication.**

Answers:

15. Would you consider an invitation, to an assessment of your English language knowledge, at the Hospital das Clínicas facility? The assessment should take approximately 60 minutes.

Yes.	
No.	

16. Should this evaluation of your English language knowledge, take place in one of the Cultura Inglesa facilities, in São Paulo, and result in a score and official certification of your current knowledge, would you be interested in participating?

Yes.	
No.	
