# Supplementary material for: Success in publication by graduate students in psychiatry in Brazil: an empirical evaluation of the relative influence of English proficiency and advisor expertise
Source: BMC Med Educ. 2014 Nov 6;14:238. doi: 10.1186/1472-6920-14-238 (PMC4289391; doi:10.1186/1472-6920-14-238)
Supplement: Supplementary file 2 — Additional file 2: List of ISI-indexed journals where students’ articles were published. (PDF 100 KB) [file 12909_2013_1058_MOESM2_ESM.pdf]

Appendix 2. List of ISI-indexed journals where students´ articles were published

Journal	Impact factor	Language the article was published	Articles per journal	Authors per article
Acta Neuropsychiatrica	0.606	english	1	4
Addictive Behaviors	2.021	english	1	2
Alcohol and Alcoholism	1.956	english	2	2;2
American Journal of Psychiatry	14.721	english	1	7
Brazilian Journal of Pharmaceutical Sciences	0.373	english	1	2
British Journal of Psychiatry	6.606	english	1	2
Canadian Journal of Psychiatry	2.483	english	1	8
Clinical Therapeutics	2.23	english	1	4
Clinics	2.058	english	2	32;6
CNS Neuroscience & Therapeutics	4.458	english	1	5
Epilepsy & Behavior	1.844	english	6	4;6;4;6;5;4
International Journal of Neuropsychopharmacology	5.641	english	1	8
Journal of Clinical and Experimental Neuropsychology	1.862	english	2	5;3
Journal of Neural Transmission	3.052	english	1	7
Journal of Psychiatric Research	4.066	english	1	8
Psychotherapy and Psychosomatics	7.23	english	1	7
Revista Brasileira de Med. Do Esporte	0.268	portuguese	1	3
Revista Brasileira de Psiquiatria	1.856	english	5	4;4;2;3;6
Revista de Psiquiatria Clínica	0.633	portuguese	4	4;4;2;3
Schizophrenia Research	4.590	english	2	6;3
World Journal of Biological Psychiatry	3.571	english	1	10
